# Pulled microcapillary tube resonators with electrical readout for mass sensing applications

**DOI:** 10.1038/srep33799

**Published:** 2016-10-03

**Authors:** Donghyuk Lee, Joonhui Kim, Nam-Joon Cho, Taewook Kang, Sangken Kauh, Jungchul Lee

**Affiliations:** 1 School of Mechanical and Aerospace Engineering, Seoul National University, Seoul, 08826, Korea; 2 School of Materials Science and Engineering, Nanyang Technological University, Singapore, 639798, Singapore; 3 Department of Chemical and Biomolecular Engineering, Sogang University, Seoul, 04107, Korea; 4 Department of Mechanical Engineering, Sogang University, Seoul, 04107, Korea

## Abstract

This paper reports a microfabrication-free approach to make hollow channel mass sensors by pulling a glass capillary and suspending it on top of a machined jig. A part of the pulled section makes simple contact with an actuation node and a quartz tuning fork (QTF) which acts as a sensing node. The two nodes define a pulled micro capillary tube resonator (P*μ*TR) simply supported at two contacts. While a piezo actuator beneath the actuation node excites the P*μ*TR, the QTF senses the resonance frequency of the P*μ*TR. The proposed concept was validated by electrical and optical measurements of resonant spectra of P*μ*TR. Then, different liquid samples including water, ethanol, glycerol, and their binary mixtures were introduced into the P*μ*TR and the resonance frequency of the P*μ*TR was measured as a function of liquid density. Density responsivity of −3,088 Hz-g^−1^ cm^3^ obtained is comparable to those of microfabricated hollow resonators. With a micro droplet generation chip configured in series with the P*μ*TR, size distribution of oil droplets suspended in water was successfully measured with the radius resolution of 31 nm at the average droplet radius, 28.47 *μ*m. Overall, typical off-the-shelf parts simply constitute a resonant mass sensing system along with a convenient electrical readout.

One of the most popular physical sensors is a mass sensor^1^,^2^,^3^,^4^,^5^ relying on resonance frequency shift of the structure. With the aid of the development of micro- and nanofabrication techniques, there are numerous efforts and ongoing progress towards performance enhancement via miniaturization of sensing devices. Nanoelectromechanical systems (NEMS) resonators have achieved zeptogram-scale mass resolution almost a decade ago^6^ and bottom-up synthesized nanomaterials based resonators have enabled single atom^7^ and proton^8^ resolution. Encouragingly, nanomechanical resonators have recently enabled single-molecule mass spectrometry in real time^9^. However, exceptional resolutions achieved with NEMS and nanomaterials based resonators are in general limited to ideal conditions such as high vacuum levels and cryogenic temperatures. Such experimental environments are far from being compatible to portable field applications and minuscule matters suspended in liquid, not to mention that the maintenance of such operating conditions requires intensive time, cost and labor.

Hollow resonators^10^,^11^,^12^,^13^,^14^ have become one of the most promising platforms for inertial sensing of liquids and particle suspensions with the hollow core intrinsically serving as a sample delivery and transport channel. The embedded channel localizes liquid samples and makes suspended particles guided towards the mass sensing region on demand. This is a unique advantage of hollow resonators over solid ones that are fully exposed to liquid sample and passively observe stochastic landing of suspended particles. Most hollow resonators reported to date have been made via sacrificial processes^10^,^13^ and fusion bonding^11^,^12^. Hollow resonators have shown numerous applications with unprecedented performance such as mass^11^,^15^,^16^,^17^,^18^,^19^, density^16^,^17^, volume^17^, surface charge^20^, and deformability^21^,^22^ measurements of single micro-/nanoparticles or cells, however, slow and complicated fabrication hampers widespread adaptation of hollow resonators. The fabrication time and complexity are further increased if an electrical readout for the resonance frequency is harnessed into hollow resonators^23^.

A novel approach by atomic migration of silicon recently reported has significantly simplified and expedited the overall fabrication of hollow resonators^24^. Nevertheless, researchers who have no or limited access to standard microfabrication facilities housed in a cleanroom hardly take advantage of the simple process. Interestingly, a commercially available microcapillary suspended over a pre-defined trench seems very promising since it is potentially available to anyone. Suspended microcapillary resonators with optical detection were employed for liquid density measurements^25^ and differentiation of X- and Y- chromosome bearing sperm cells^26^. Despite the absence of commercially available nanoscale capillary tubes, suspended nanocapillary resonators^27^ have been simply fabricated by using polymer nanofibers deposited over lithographically pre-defined trenches as sacrificial templates and used with an optical interferometry. Optical detection, however, makes the overall system bulky and requires a precise alignment. In addition, small inner and outer diameters of micro-/nanocapillaries are not directly compatible with existing standard fluidic tubings and connectors.

Here, we introduce an innovative solution for aforementioned issues—a hollow microchannel mass sensing system which exhibits user friendly fluidic interconnection and alignment-free electrical readout by using pulled glass microcapillaries and quartz tuning fork (QTF) without relying on any conventional microfabrication process. After the proposed system was thoroughly tested and validated, mass densities of various liquid samples and sizes of oil microdroplets suspended in water were measured with the responsivity comparable to microfabricated hollow resonators.

## Results

### Materials and fabrication

**Figure 1:**
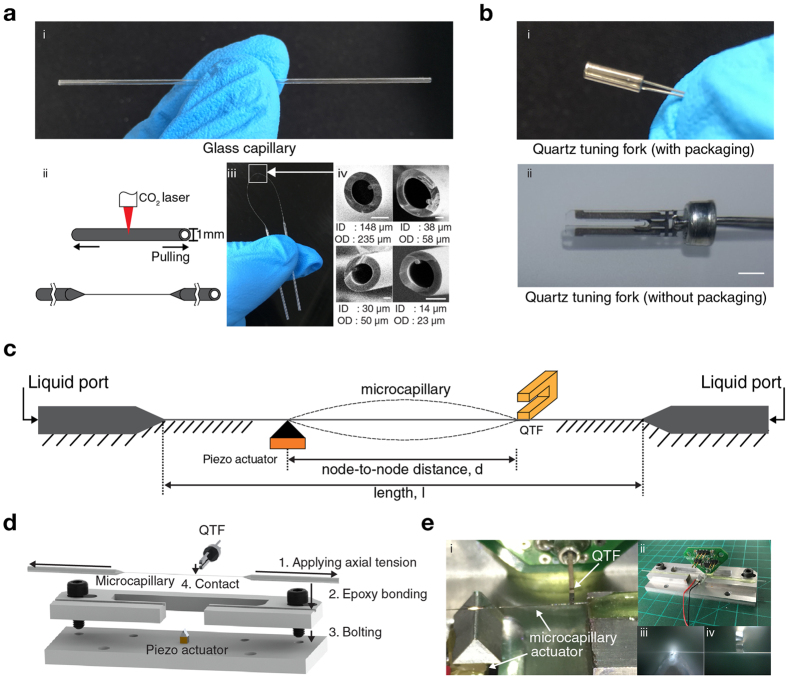
Off-the-shelf glass capillary and quartz tuning fork (QTF) components simply modified and assembled toward a microfabrication-free microscopic sensing platform. (**a**) A glass capillary with inner and outer diameters of 0.58 and 1.0 mm, respectively (top). Schematic showing the capillary pulling to decrease the inner and outer diameters around the middle section of the capillary (bottom left). A pulled capillary (bottom middle). Scanning electron micrographs of cross-section areas of pulled capillaries with various inner and outer diameters. Scale bars are 100, 10, 10, and 10 *μ*m, respectively (bottom right). (**b**) A QTF before (top) and after (bottom) the package is opened and removed. The scale bar is 1 mm. (**c**) A pulled micro capillary tube resonator (P*μ*TR) of which one nodal point is defined by a physical contact to one of two prongs of a QTF. (**d**) Assembly process of P*μ*TR-QTF system (not to scale). (**e**) Photographs of an assembled P*μ*TR-QTF sensing platform ready for testing.

To fabricate microcapillary tube resonators without relying on conventional microfabrication in a cleanroom, we employed a capillary pulling process with a borosilicate glass capillary of which inner and outer diameters are 0.58 and 1.00 mm, respectively, (Fig. 1a(i)) (1B100F-3, World Precision Instruments) and a commercial pipette puller (P-2000, Sutter Instrument). The laser-heated pipette puller defines the microcapillary tube through sequential heating and pulling processes which locally stretch the middle section of the glass capillary while maintaining the cross-sectional area of both ends (Fig. 1a(ii)). This is somewhat different from the typical use of a pipette puller which is used to produce two symmetric pipettes with a nanoscale fluidic access hole. Once the glass capillary was partially pulled, it was flexible enough to be bent 180 degree and make a U-shaped loop (Fig. 1a(iii)). Furthermore, it could be wound up and make a 360-degree loop as shown in Fig. S1. Inner and outer diameters of the pulled section of the glass microcapillary can be varied by adjusting parameters such as laser power and pulling force. The smallest and largest inner diameters of the pulled section were 14 and 148 *μ*m, respectively, while the ratio of inner to outer diameter of the pulled section was 0.623 ± 0.025 which is similar to the initial ratio of 0.58 (Fig. 1a(iv)). Hereafter, pulled microcapillaries of which inner and outer diameters are 30 and 50 *μ*m and overall elongation length is 58 mm were used.

For simple and robust sensing of the resonance frequency of pulled microcapillary tubes, a QTF (AB26T-32.768 kHz, Abracon) was employed after its hermetic metal can packaging was removed. Figure 1b(i,ii) show the QTF before and after the removal of the hermetic packaging, respectively. For actuation of pulled microcapillary tubes, a piezo actuator (TA0505D024W, Thorlabs) was employed.

Figure 1c illustrates the overall schematic of the pulled microcapillary tube resonator (P*μ*TR). A microcapillary tube with axial tension applied makes contact with a solid block on top of a piezo actuator and one prong of a QTF. The contact point above the piezo actuator becomes the actuation node while the contact point with the QTF becomes the sensing node. The node-to-node distance (i.e. distance between two contact points) defines the length of the (P*μ*TR). Unpulled sections at both ends facilitate interfacing with standard fluidic components. To realize the proposed P*μ*TR, parts were assembled by following the procedures in Fig. 1d. First, 0.3 *N* of axial tension was applied to a pulled microcapillary using a tension gauge (110 g, OHBA SIKI) to remove slacks and also increase the resonance frequency of P*μ*TR. Effect of the applied tension on the amplitude signal and maximum axial tension allowed can be found in Supplementary information section 1 (Figs S2 and S3). With the applied tension maintained, the pulled microcapillary was brought over and attached to a custom machined aluminum jig with an epoxy. Then, the jig holding the attached microcapillary was fixed to a base block having a nodal block on the piezo actuator with two standard M4 bolts. After this step, the pulled microcapillary made a solid contact with the actuation node. Finally, using a motorized stage (MTS25-Z8, Thorlabs), the QTF mounted on a custom printed circuit board (PCB) approached precisely with a 10-*μ*m step towards the pulled microcapillary and made another solid contact which defined the sensing node. Finally, the PCB was bonded to the jig assembly by using the epoxy. Figure 1e shows several pictures of the final P*μ*TR-QTF coupled system. The typical setting for the node-to-node distance was 6.5 mm throughout this work unless otherwise stated.

### Theory and operation

**Figure 2:**
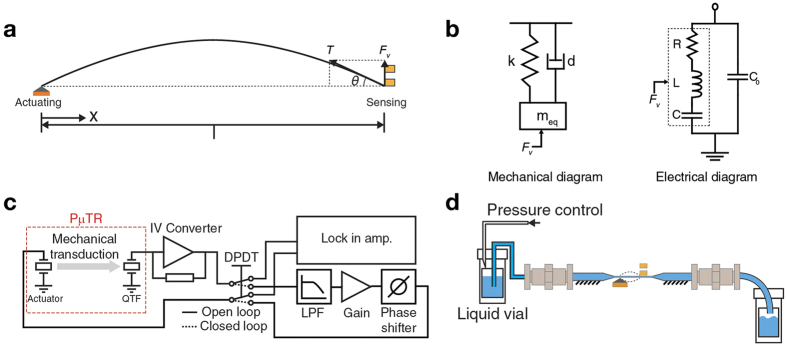
Operation scheme of the P*μ*TR-QTF coupled sensing system. (**a**) Free body diagram for the P*μ*TR-QTF. (**b**) Mechanical and electrical equivalent models for the QTF which generates electrical currents via piezo-electric transduction of the vertical reflection force, *F_v_*. (**c**) Open and closed loop drive circuits for the P*μ*TR-QTF system. (**d**) Fluidic setup diagram to deliver a liquid sample into the P*μ*TR.

The resonance frequency, *f*, of the P*μ*TR under axial tension, *T*, is given by^28^


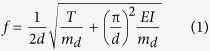


where *d* is the P*μ*TR’s length, *E* is the Young’s modulus, *I* is the area moment of inertia, and *m*_*d*_ is the mass per unit length of the P*μ*TR, respectively. When the operation of the actuating node induces out-of-plane vibration of the P*μ*TR, the vertical reflection force, *F*_*v*_, which is the vertical component of the applied axial tension, is felt by the QTF and is given by


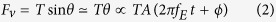


where *θ* is the tangent angle of the P*μ*TR at the QTF sensing node, *A* is the vibration amplitude, *f*_*E*_ is the excitation frequency, *t* is time, and *ϕ* is the phase delay (Fig. 2a). This vertical reflection *F*_*v*_, of which magnitude is proportional to the vibration amplitude of the P*μ*TR is transmitted to the QTF, then, induces a forced vibration at one of two prongs with the frequency of *f_E_*. Although the proposed actuation scheme is asymetric, the response from the P*μ*TR is symmetric (Fig. S4d). Dynamic amplitude of the equivalent spring-mass-damper system for the QTF with an external input of *F*_*v*_ is transduced to dynamic current generation at the RLC components in a Van-Dyke equivalent circuit due to the intrinsic piezoelectric effect of the QTF^29^ (Fig. 2b). A transimpedance amplifier was configured using an operational amplifier (OPA129, Texas Instruments) with a 200 kΩ gain resistor and connected to one electrode of the QTF for current-to-voltage conversion with a gain while the other electrode of the QTF was grounded.

The P*μ*TR-QTF system can be operated in either open or closed loop modes (Fig. 2c). In the open loop mode, a function generator drives the piezocrystal actuator at the actuation node near the resonance frequency of the P*μ*TR while the piezoelectrically transduced vibration of the P*μ*TR by the QTF sensing node is measured with a lock-in amplifier (Model 7265, Signal Recovery) through the aforementioned transimpedance amplifier. In the closed loop mode, the output from the transimpedance amplifier is low pass filtered, amplified, phase-shifted and fed back to the piezo actuator at the actuation node. The filter used is a second-order active low-pass filter (LPF) with the cut-off frequency of 30 kHz (Voltage pre-amplifier SR560, Stanford Research Systems). For sustaining oscillation of the P*μ*TR, the insertion of the LPF is crucial for the piezoelectric readout where two distinct resonance peaks with comparable amplitudes are present (see Open loop operation Section for details). For real-time tracking of the resonant frequency of the P*μ*TR, the feedback signal is mixed-down to approximately 1 kHz by an analog multiplier (AD734, Analog Devices) and measured by using a 100 MHz frequency counter equipped in the data acquisition board (USB-6361, National Instruments). For both open and closed loop operations, the voltage signal fed into the piezo actuator at the actuation node is restricted within ±15 V in order to prevent excessive oscillation amplitude of the P*μ*TR which may affect the contact status between the actuation node and P*μ*TR and induce problems associated with nonlinearities such as bi-stabilities. For our entire measurements, the vibration amplitudes are far below the onset of nonlinearity (see Supplementary information section 2) and the contact status was stable. Even at the maximum drive condition in our current experimental setup, we were not able to get into the nonlinear dynamics regime. We have not seen any noticeable changes in phase spectra as the drive amplitude was varied (Fig. S5). This is also because the P*μ*TR was operated in the linear regime.

To introduce liquid samples into the P*μ*TR, a pneumatic system was installed with electronic pressure regulators (ITV-0030, SMC), pressurizable vials, standard tubings (1/16″ FEP, Upchurch Scientific) and standard connectors as shown in Fig. 2d. Such interfacing with standard tubings and connectors is a unique advantage of the P*μ*TR over fluidic resonators based on microcapillary tubes or microfabricated hollow resonators.

### Open loop operation

**Figure 3:**
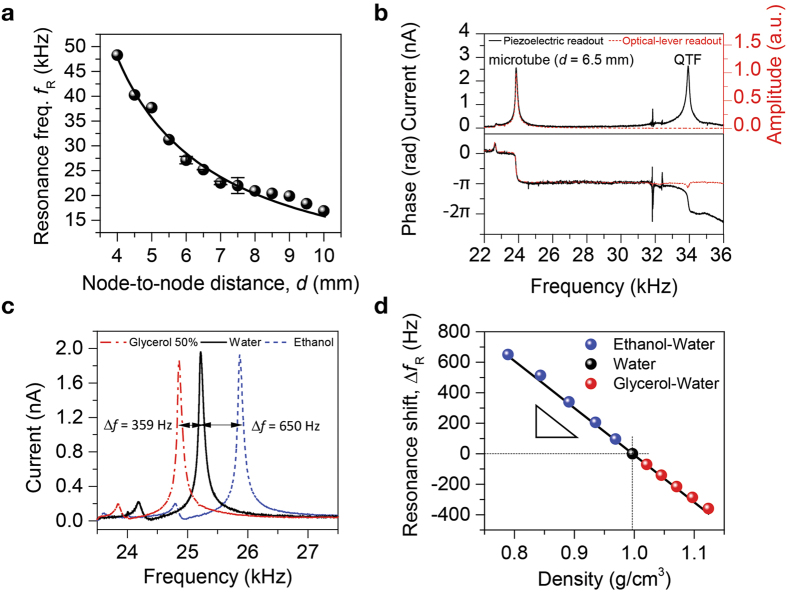
Open loop operation of the P*μ*TR-QTF system intended for density calibration and measurements. (**a**) Resonance frequency as a function of node-to-node distance (or length) of the P*μ*TR filled with water. (**b**) Amplitude (top) and phase (bottom) spectra of a water filled 6.5-mm long P*μ*TR measured by optical-lever (red dashed line) and piezoelectric (solid black line) readouts. While the resonance peak for the P*μ*TR around 24.5 kHz is universally detected, the QTF resonance around 33 kHz is exclusively measured by the piezoelectric readout. (**c**) Resonant spectra of a 6.5-mm long P*μ*TR filled with water, ethanol, and glycerol-water mixture (glycerol:water = 1:1 in weight ratio). (**d**) Resonance frequency shift of the 6.5-mm long P*μ*TR measured while water, ethanol-water binary mixtures, and glycerol-water binary mixtures are sequentially introduced. The density responsivity of −3,088 Hz g^−1^ cm^3^ is obtained from the linear regression.

For proof-of-concept experiments, a couple of basic measurements were conducted in the open loop operation mode. After P*μ*TRs with various lengths were prepared by adjusting the node-to-node distance and filled with water, their resonance frequencies were measured sequentially using the QTF sensing node via piezoelectric transduction and plotted as a function of the node-to-node distance (Fig. 3a). Their amplitude spectra and quality factors can be found in Fig. S6. Measurements show good agreement with theoretical calculation, the solid line in Fig. 3a, by Equation (1). To validate the piezoelectric QTF sensing mechanism, a complementary optical-lever measurement was performed for a 6.5-mm long P*μ*TR with a partially deposited metal (gold) reflector (see Supplementary information section 3 and Fig. S7a). While the piezoelectric readout captures two resonance peaks, one near 24.5 kHz for P*μ*TR and the other near 33.0 kHz for QTF, the optical-lever readout measures a single resonance peak at 24.5 kHz for P*μ*TR (Fig. 3b). This difference in two readout schemes is explained in detail by the block digram (see Supplementary information section 3 and Fig. S7b,c). The resonance peak for the P*μ*TR was universally detected by two different readout schemes. Therefore, the expensive and bulky optical setup which accompanied an additional metal coating for reflective laser signal and precise alignment is circumvented once the simple QTF sensing node based on piezoelectric transduction. Moreover, the piezoelectric readout can be superior to the optical-lever readout in terms of the frequency stability unless significant amount of effort is made towards precise optical alignment or high-end optical components are used (Fig. S8).

Next, another 6.5-mm long P*μ*TR was prepared and its resonance frequency was measured by using the QTF sensing node while water, ethanol, and glycerol-water mixture (glycerol:water = 1:1 in weight ratio) were sequentially introduced into the P*μ*TR (Fig. 3c). Then, for more precise calibration, various ethanol-water and glycerol-water binary mixtures were prepared and injected in the P*μ*TR while the resonance frequency of P*μ*TR was measured (Fig. 3d). From the linear regression of the frequency shift vs. density plot, the density responsivity of −3,088 Hz-g^−1^ cm^3^ was extracted at room temperature.

### Closed loop operation

**Figure 4:**
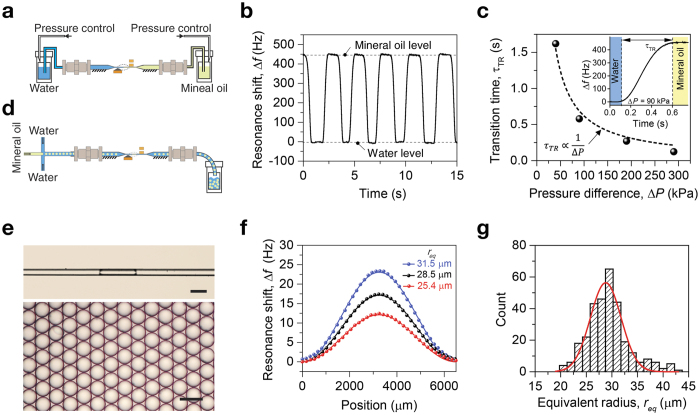
Closed loop operation of the P*μ*TR-QTF system intended for real-time applications. (**a**) Fluidic configuration modified for switching between two immiscible liquid samples. (**b**) Resonance frequency shift measured at 1-kHz bandwidth and the pressure difference of 90 kPa while water and mineral oil alternately fills the pulled micro capillary tube. (**c**) Transition time, *τ*_*TR*_, from water to mineral oil as a function of the pressure difference, Δ*P*, showing the expected inverse relationship. Inset shows a typical transition at the pressure difference of 90 kPa. (**d**) A micro droplet generator configured at the inlet of the P*μ*TR for real-time monitoring of droplet size. (**e**) Optical micrographs of a squeezed oil plug passing through the P*μ*TR (top) and collected spherical droplets after measurements (bottom). Scale bars in both images are 100 *μ*m. (**f**) Resonance frequency shifts fitting upon a transit of mineral oil plugs of which equivalent radii are 25.4, 28.5 and 31.5 *μ*m, respectively. (**g**) Histogram for the equivalent radius of oil droplets (cumulative counts are 310).

With the closed loop operation mode shown in Fig. 2c, the event which occurs at a much faster time scale can be examined with the P*μ*TR-QTF system. First, the fluidic configuration shown in Fig. 4a was implemented. While deionized (DI) water was filled in one pressurizable vial, mineral oil (Mineral oil light, Sigma Aldrich) was loaded in another pressurizable vial. Due to the immiscible nature of two liquid samples, the water-oil interface was maintained. By alternating the pressure difference of 90 kPa between two vials, a transition from DI water to mineral oil or vice versa could be monitored as shown in Fig. 4b. The difference in the resonance frequency of P*μ*TR due to the water-mineral oil exchange was approximately 450 Hz which corresponds to 0.851 g/cm^3^ based on the calibration shown in Fig. 3d. This is in good agreement with the specification, 0.838~0.854 g/cm^3^ (25 °C) given by the supplier. As expected, the transition time from water to mineral oil was found to be a function of the pressure difference, *τ*_*TR*_ ∝ Δ*P*^−1^. Second, a microscale droplet generator was configured at one entrance of the P*μ*TR as shown in Fig. 4d. The interior of the droplet generator was hydrophilic so mineral oil was used as a disperse phase and water was used as a continuous phase (i.e. mineral oil droplets in water). In the water, a surfactant (Tween20, Sigma Aldrich) was mixed at a volume fraction of 0.5 percents. Pressure settings for the droplet generator were set to produce oil droplets of which mean radii are approximately 28 *μ*m. When a oil droplet passed through the P*μ*TR, it was squeezed to form a plug since its diameter is larger than the inner diameter of the P*μ*TR as shown in Fig. 4e (top). Once they came out of the pulled section of the P*μ*TR, their shapes restored back to spheres and collected in the outlet vial (Fig. 4e (bottom)). Since the length of squeezed plugs are in general much shorter than the node-to-node distance, the assumption of a point mass traveling through the long string resonator is valid. Figure 4f shows resonance frequency shifts of the P*μ*TR recorded when oil plugs with three different lengths passed through the P*μ*TR. To quantify equivalent radii of oil plugs, buoyant mass of each plug was first calculated from fitting measured resonance shift data with the theoretical estimations given by^30^


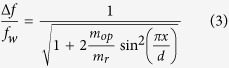


where Δ*f* is the resonance frequency shift due to an oil plug transiting the P*μ*TR, *f*_*w*_ is the resonance frequency of the P*μ*TR entirely filled with water, *m*_*op*_ is the buoyant mass of the oil plug, *m*_*r*_ is the mass of the P*μ*TR, and *x* is the lateral position of the traveling oil plug. The simple harmonic mode shape is valid for P*μ*TR longer than 5 mm (Fig. S10). Then, an equivalent spherical radius, *r*_*eq*_, can be given by


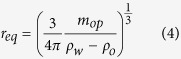


where *ρ*_*w*_ and *ρ*_*o*_ are densities of water and mineral oil, respectively. When either the buoyant mass or the equivalent radius of an oil plug are extracted, its velocity is simultaneously obtained (see Methods and Supplementary information section 5). Measurements were continued for several hundreds of oil plugs and a histogram of the equivalent radius was acquired with the average of 28.47 *μ*m and the standard deviation of 4.03 *μ*m (Fig. 4g). Simultaneously obtained velocity histogram and velocity vs. radius scatter plot are shown in Fig. S12. Interestingly, spherical oil droplets collected after measurements show a uniform distribution (Fig. 4e (bottom)). Unless a higher magnification is employed, small difference in the droplet size can not be easily observed. Even with a higher magnification, conventional far-field optical measurements are diffraction limited. The P*μ*TR-QTF system truly offers more precise and more quantitative measurements for droplet sizing than general optical microscopy. In addition, the P*μ*TR-QTF system exclusively provides density and mass of droplets.

### Performance metrics of P*μ*TR systems

**Figure 5:**
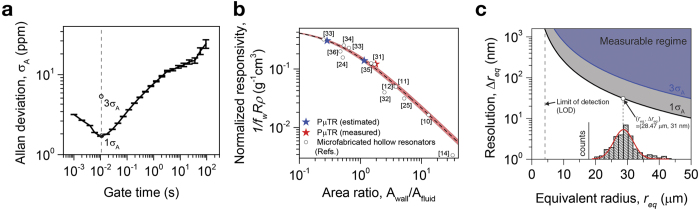
Frequency noise and density responsivity of the P*μ*TR-QTF system. (**a**) Allan deviation of the 6.5-mm long P*μ*TR with water filled as a function of gate time. The piezoelectric readout is used. The best frequency stability is obtained at the gate time of 0.01 s. (**b**) Normalized density responsivity of the 6.5-mm long P*μ*TR in comparison with those of other micro fabricated density sensors found from literatures plotted (band plot) as a function of area ratio (density of wall, *ρ*_*wall*_, is set to be 2.25 ± 0.25 g/cm^3^). The dashed line along with pink shade shows the theoretically estimated responsivity. (**c**) Detection resolution of the difference in mineral oil droplet radius as a function of the equivalent oil droplet radius (calculated from frequency stabilities, 1 and 3 *σ*_*A*_). Figure 4g is included as inset figure to show the radius distribution of measured oil plugs. The limit of detection (LOD) for the smallest droplet radius is 4.25 *μ*m.

Figure 5a shows the Allan deviation *σ*_*A*_ of the normalized resonance frequency of the P*μ*TR filled with water as a function of the gate time (Fig. S8 to compare Allan deviations from the piezoelectric and optical-lever readouts). While the water-filled P*μ*TR was operated in closed loop, its resonance frequency was sampled and recorded approximately every 1 ms. As the gate time increases, the Allan deviation measured decreases, reaches a minimum around 0.01 s, and increases. Therefore, the best frequency stability 1.66 ppm was obtained at the gate time of 0.01 s. This frequency stability is only one order of magnitude lower than that of the microfabricated hollow channel resonator with integrated piezoresistive readout^23^.

Normalized density responsivity of our P*μ*TR-QTF system was plotted as a function of the ratio of resonator wall cross-sectional area to channel cross-sectional area (area ratio) and compared with those of other density sensors based on microfabricated hollow resonators^10^,^11^,^12^,^14^,^24^,^25^,^31^,^32^,^33^,^34^,^35^,^36^ (Fig. 5b). The solid line in Fig. 5b is theoretical estimation by


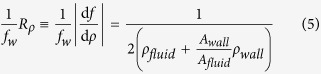


where *f*_*w*_ and *f* are resonance frequencies of a P*μ*TR filled with water and actual sample, respectively, *ρ*_*fluid*_ and *ρ*_*wall*_ are densities of the fluid sample and resonator wall material, respectively, and *A*_*wall*_ and *A*_*fluid*_ are cross-sectional areas of the resonator wall and channel, respectively. Normalized density responsivity of our P*μ*TR-QTF system is better than several microfabricated hollow channel density sensors (Fig. S9 for data from three P*μ*TR-QTF pairs). With different to particle mass (i.e. point mass) responsivity inversely proportional to the resonator’s effective mass, normalized density responsivity depends on the area ratio, *A*_*wall*_/*A*_*fluid*_ only (Equation (5)). Therefore, miniaturization does not necessarily improve the normalized density responsivity. Once a thinning process of the resonator wall such as the selective exterior etching is fully developed, the normalized density responsivity of the P*μ*TR-QTF system can be further improved. For example, the outer wall of the pulled section can be exposed to and etched in glass etching solutions such as hydrofluoric acid and buffered oxide etch solution thus the area ratio can be decreased (see Supplementary information section 6 and Fig. S13), with the aid of the flexibility which offers 180 degree bending or 360 degree wound up loop shown in Fig. 1a and Fig. S1, respectively. However, after the selective exterior etching, P*μ*TR became very fragile thus easily broken with the piezoelectric readout which requires hard contact between the microcapillary and the QTF for the etched P*μ*TR of which wall thickness is less than 4 *μ*m. We suggest that the proposed actuation scheme would be paired with the capacitive detection with a remote sensing electrode or with the optical detection which was already used for proof-of-concept experiments (see Supplementary information section 3).

Figure 5c shows the detection resolution of the difference in mineral oil droplet radius, Δ*r*_*eq*_, plotted as a function of the equivalent oil droplet radius. This resolution was calculated from frequency stabilities, 1 and 3 *σ*_*A*_ (see Equation (8) in the Methods section). For the average droplet radius of 28.47 *μ*m shown in Fig. 4g, we can differentiate oil droplets if their radius differ by approximately 31.66 and 94.99 nm based on the 1 and 3 *σ*_*A*_ criteria, respectively. This resolution is far superior to the diffraction limit of general optical microscopy based techniques. As the droplet gets smaller, the resolution of Δ*r*_*eq*_ becomes negatively affected. The colored area above each solid line represents the “Measurable regime” based on each criterion. The limit of detection in the equivalent oil droplet radius (i.e. the smallest oil droplet to be measured) is 4.25 *μ*m.

## Discussion

Here, we proposed a microfabrication-free approach to realize microscale fluidic resonators for gravimetric sensing applications by configuring three off-the-shelf parts; glass capillary, piezocrystal actuator, and quartz tuning fork. Commercially available glass capillary with a millimeter scale diameter were pulled to reduce their diameters to several tens of micrometers and increase overall lengths by a few centimeters via laser heating. As a result, microcapillary tubes were locally defined in the middle of glass capillary while two ends maintained original millimeter scale dimensions. Fabricated microcapillary tubes were mounted on a custom machined jig and made contact with two nodes to complete the fabrication process. One contact point was the actuation node, a machined aluminum block on a piezocrystal actuator which applied out-of-plane vibration. The other contact point was the sensing node, a prong of a commercially available QTF. This P*μ*TR-QTF hybrid system offers the following benefits; simple fabrication of microfluidic resonators, electrical readout of the resonance frequency and facile connection to standard tubings and fluidic components.

After manufacturing the P*μ*TR-QTF hybrid system, two gravimetric applications were demonstrated. In the first application, the P*μ*TR-QTF hybrid system was operated in open loop to sense the mass density of water, ethanol, glycerol and their binary mixtures introduced into the P*μ*TR. In the second application, the system was operated in closed loop to weigh and size mineral oil droplets passing through the P*μ*TR. With the Allan deviation separately acquired, we found out that the smallest radius mineral oil droplet measurable is 4.25 *μ*m.

Encouragingly, the normalized density responsivity of our P*μ*TR-QTF hybrid system is higher than a group of microfabricated hollow channel density sensors. Diameters and wall thickness of the P*μ*TR can be further downsized by optimization of the pulling process or selective etching from either inner or outer surfaces. The structural material of the P*μ*TR is transparent to visible light so that many optical and spectroscopic techniques can be easily combined with the P*μ*TR. In addition, other capillary experimental setup based separation and detection schemes such as capillary electrophoresis^37^ and whispering gallery mode optical cavity^38^ can be integrated to realize micro total analysis systems or multimodal sensing platforms.

## Methods

### Data processing for equivalent radius and velocity of oil plugs

Frequency change data due to traveling oil plugs recorded in time were processed to extract equivalent radius and velocity of oil plugs as illustrated in Fig. S11. First, recorded raw data were Savitzky-Golay filtered (window of 50 and polynomial order of 3). Then, first time derivatives of the frequency change were compared with a pre-configured threshold to locate each peak resulting from an oil plug. For each selected peak, a small window was masked to get a baseline to be compensated. With the node-to-node distance (or resonator length) and the constant velocity assumption of traveling oil plugs, filtered and baseline compensated frequency change data were fitted by Equation (3) to obtain the buoyant mass and velocity of oil plugs. The buoyant mass was converted to the equivalent radius by Equation (4) where densities of water and oil separately measured with the P*μ*TR were used. The constant velocity assumption for oil plugs traveling through the P*μ*TR is valid because oil plugs do not exhibit motion relative to the surrounding water and the vibration amplitude of the P*μ*TR is negligibly small.

### Calculation for resolution of the difference in oil plug equivalent radius

The detection resolution of the difference in equivalent radii of oil plugs 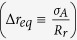 is obtained by dividing the Allan deviation (*σ*_*A*_) of the P*μ*TR from the radius responsivity 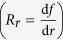. The chain rule is applied to the radius responsivity to include the buoyant mass of oil plugs (*m*_*op*_) as follows.


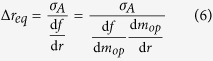


The derivative of 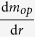 derived by spherical mass-volume relationship 
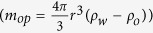
 is given by


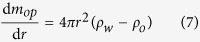


Finally, by employing Equations (6) and (7), the relationship between the mass and radius resolutions is obtained as


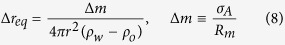


The mass responsivity 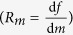 is acquired by fitting data of transiting oil plugs shown in Fig. 4f,g. and used here. Equation (8) indicates that the radius resolution is inversely proportional to the square of its own radius.

## Additional Information

**How to cite this article**: Lee, D. *et al.* Pulled microcapillary tube resonators with electrical readout for mass sensing applications. *Sci. Rep.* **6**, 33799; doi: 10.1038/srep33799 (2016).

## Supplementary Material

Supplementary Information
