# Supplementary material for: Pulled microcapillary tube resonators with electrical readout for mass sensing applications
Source: Sci Rep. 2016 Oct 3;6:33799. doi: 10.1038/srep33799 (PMC5046181; doi:10.1038/srep33799)
Supplement: Supplementary Information [file srep33799-s1.pdf]

Supplementary information: Pulled microcapillary tube resonators with electrical readout for mass sensing applications

Donghyuk Lee[1,+], Joonhui Kim[2,+], Nam-Joon Cho[2], Taewook Kang[3], Sangken Kauh[1], and Jungchul Lee[4,*]

[1]School of Mechanical and Aerospace Engineering, Seoul National University, Seoul, 08826, Korea
[2]School of Materials Science and Engineering, Nanyang Technological University, Singapore, 639798, Singapore
[3]Department of Chemical and Biomolecular Engineering, Sogang University, Seoul, 04107, Korea
[4]Department of Mechanical Engineering, Sogang University, Seoul, 04107, Korea
[*]Corresponding author e-mail: jayclee@sogang.ac.kr

Contents

1 Characterization for assembly of the PμTR **3**

2 Characterization for operation of the PμTR **5**

3 Piezoelectric vs. optical-lever readouts **8**

4 Normalized density responsivities of PμTRs **10**

5 Analysis for oil droplet measurements **11**

6 Performance tuning for PμTRs **13**

List of Figures

1 **Flexibility of the pulled microcapillary.** A photograph showing a wound up microcapillary (left) and an optical micrograph of the zoomed-in loop showing the minimum curvature, r_{min}, of 780 μm (right). Scale bar is 1 mm. 3

2 **Effect of the applied tension on the amplitude signal coupling.** **(a)** Schematic showing how to apply the axial tension before the permanent fixation with epoxy. **(b)** Resonance frequency of the PμTR and amplitude at resonance measured by the QTF as a function of the axial tension applied. 3

3 **Maximum axial tension allowed for the PμTR as a function of its wall thickness.** The inner diameter of the PμTR is fixed to be 30 μm. The dashed line represents the control resolution of the tension gauge used in this work. 4

4 **Amplitude calibration of the PμTR.** **(a)** Schematic for atomic force microscope cantilever based calibration of local vibration amplitude of the PμTR. **(b)** Amplitude spectra acquired at the mid-point ($x = 3$ mm) of the microcapillary. **(c)** Current vs. amplitude calibration and noise floor of the piezoelectric readout. **(d)** Local amplitudes of the microcapillary measured at several lateral positions and their sinusoidal fits with various actuation voltages. **(e)** Amplitudes at the piezo actuation node ($x = 0$ mm) and the mid-point ($x = 3$ mm) of the microcapillary and their ratios as a function of the actuation voltage. 5

5 **Resonance spectra of a PμTR for several different drive amplitudes.** Amplitude (top) and phase (bottom) spectra. Four phase spectra are almost identical. The length of the PμTR is 6.5 mm. 5

6 **Resonant characteristics of PμTRs. (a)** Amplitude spectra and **(b)** quality factors of PμTRs of which lengths range from 4 to 10 mm. Error bars represent standard errors of the means with $N = 3$. 7
7 **Signal transfers in the PμTR-QTF system and its resonance spectra by optical-lever and piezoelectric readouts. (a)** Experimental schematic for optical-lever readout of PμTR with partial metal coating. **(b)** Block diagram for the PμTR-QTF system. **(c)** Amplitude and phase spectra of individual components and combined PμTR-QTF system obtained by optical-lever and piezoelectric readouts. 8
8 **Comparison the frequency stabilities between piezoelectric and optical-lever readouts.** Allan deviations of the PμTR with the length of 6.5 mm measured with piezoelectric and optical-lever readouts. Inset shows frequency stabilities of the PμTR measured in time domain. 9
9 **Normalized density responsivity of fabricated PμTRs as a function of the area ratio** . . 10
10 **Structural models for PμTRs.** Resonance frequency as a function of the length of resonator based on beam, string, and beam with axial tension models. Measurements for PμTRs of which lengths range from 4 to 10 mm are also shown. 11
11 **Data fitting processing for oil plugs to extract buoyant mass (or equivalent radius) and velocity. (a)** Frequency change vs. time raw data. **(b)** Step-by-step data processing procedure. . . . 12
12 **Velocity histogram and velocity-radius scatter plot. (a)** Histogram for the velocity of oil droplets (cumulative counts are 310). **(b)** Scatter plot of velocity versus equivalent radius of oil plugs when pressure difference of 300 kPa is applied. 12
13 **Decreasing the outer diameter (wall thickness) of PμTR via selective hydrofluoric acid etching. (a)** Schematics of the selective etching process by exposing the exterior of the PμTR to hydrofluoric acid. **(b)** Scanning electron micrographs of PμTRs before and after the selective etching. All scale bars are 50 μm. **(c)** Estimated normalized density responsivity of etched PμTRs having a same inner diameter of 30 μm and different wall thicknesses of 2, 7, and 11 μm. Inset table summarizes inner and outer diameters, wall thicknesses, and area ratios of the three PμTRs shown in (b). 13

List of Tables

1 Actuation and detection limit of the PμTR-QTF system. 6
2 Geometric dimension, tension, and characteristics of three representative PμTRs. 10
3 Equations for the resonance frequency based on the three structural models. 11

1 Characterization for assembly of the PμTR

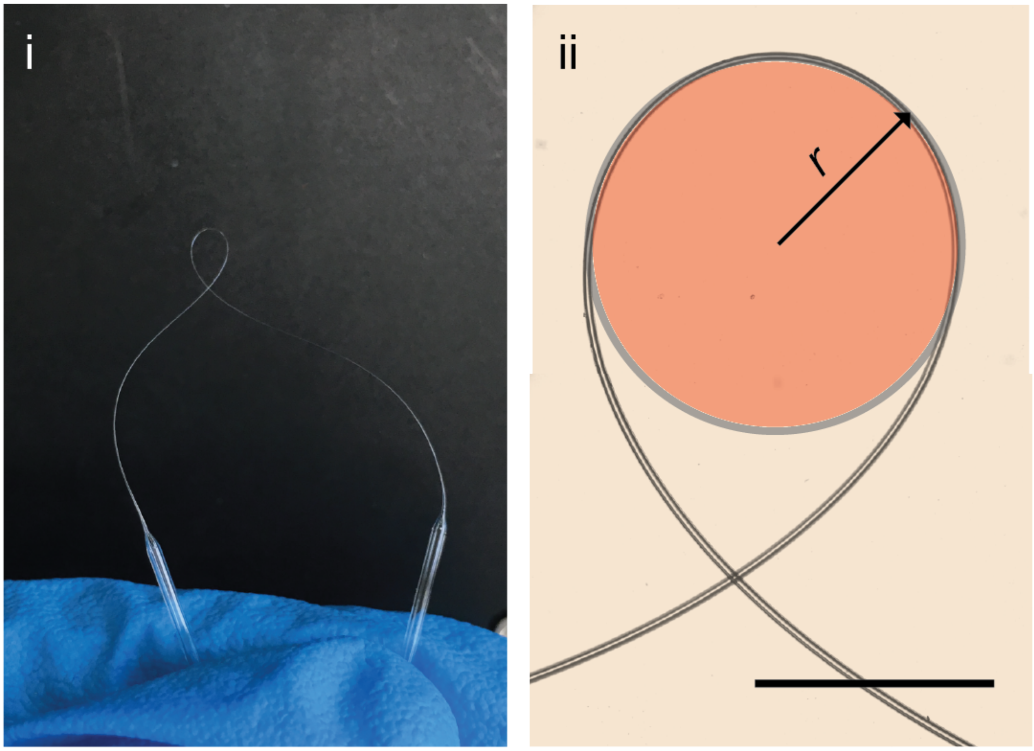

Fig. S 1: Flexibility of the pulled microcapillary. A photograph showing a wound up microcapillary (left) and an optical micrograph of the zoomed-in loop showing the minimum curvature, r_{min}, of 780 μm (right). Scale bar is 1 mm.

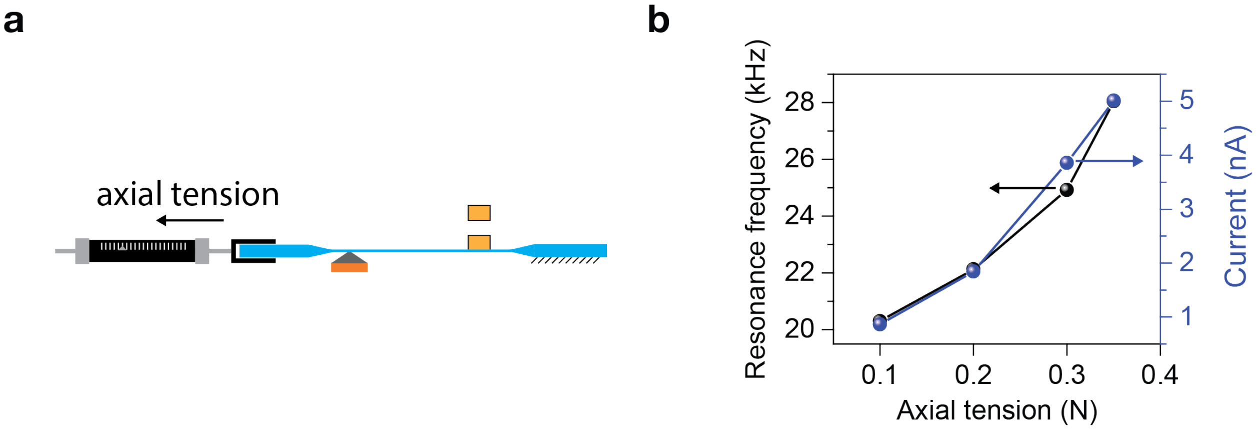

Fig. S 2: Effect of the applied tension on the amplitude signal coupling. (a) Schematic showing how to apply the axial tension before the permanent fixation with epoxy. **(b)** Resonance frequency of the PμTR and amplitude at resonance measured by the QTF as a function of the axial tension applied.

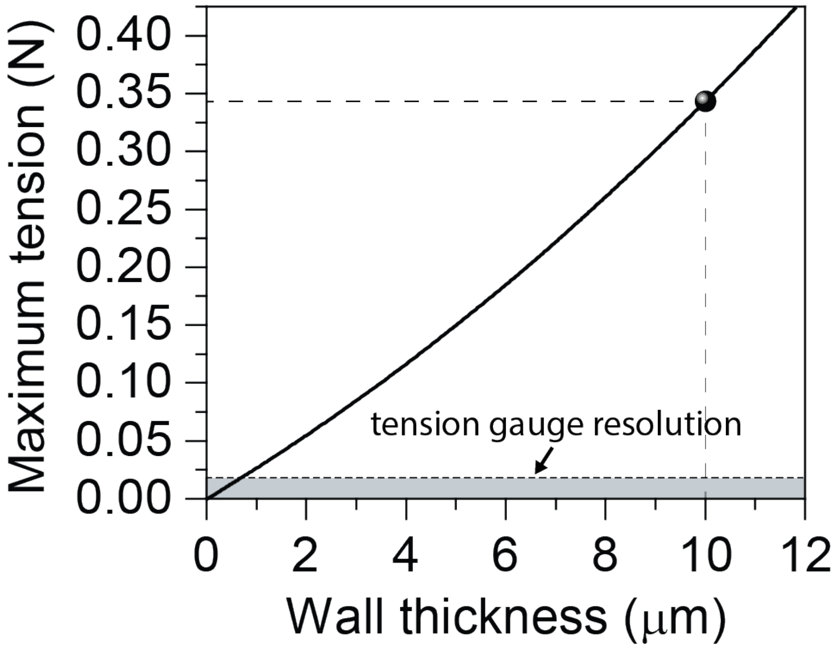

Fig. S 3: **Maximum axial tension allowed for the PμTR as a function of its wall thickness.** The inner diameter of the PμTR is fixed to be 30 μm. The dashed line represents the control resolution of the tension gauge used in this work.

Pulled microcapillaries have the aspect ratio (i.e. length-to-diameter ratio) of ∼130. They are flexible enough to be wound up to have a curvature of ∼780 μm when the inner and outer diameters are 30 and 50 μm, respectively (Fig. S1).

After the pulled microcapillary was placed on top of the machined aluminum jig, axial tension was applied to the microcapillary by using the tension gauge (110g, OHBA SIKI) (Fig. S2a). The tension preventing slacks of the microcapillary improves the mechanical coupling between the microcapillary and the QTF. As the tension increases, the amplitude measured by the QTF also increases (Fig. S2b). However, there is a limit for the axial tension that can be applied to a given microcapillary. The maximum tension allowed is limited below a threshold which is determined by the ultimate tensile strength of the borosilicate and cross-sectional area of the microcapillary[1] (Fig. S3). For a microcapillary of which inner and outer diameters are 30 and 50 μm, respectively, the maximum tension is ∼0.35 N.

2 Characterization for operation of the PμTR

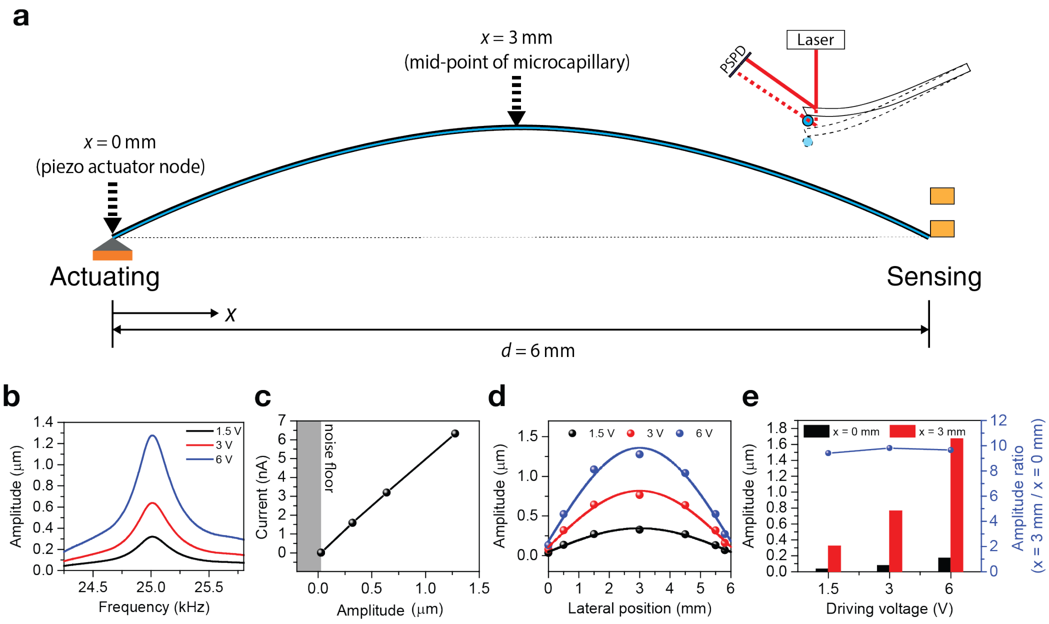

Fig. S 4: Amplitude calibration of the PμTR. (a) Schematic for atomic force microscope cantilever based calibration of local vibration amplitude of the PμTR. **(b)** Amplitude spectra acquired at the mid-point ($x = 3$ mm) of the microcapillary. **(c)** Current vs. amplitude calibration and noise floor of the piezoelectric readout. **(d)** Local amplitudes of the microcapillary measured at several lateral positions and their sinusoidal fits with various actuation voltages. **(e)** Amplitudes at the piezo actuation node ($x = 0$ mm) and the mid-point ($x = 3$ mm) of the microcapillary and their ratios as a function of the actuation voltage.

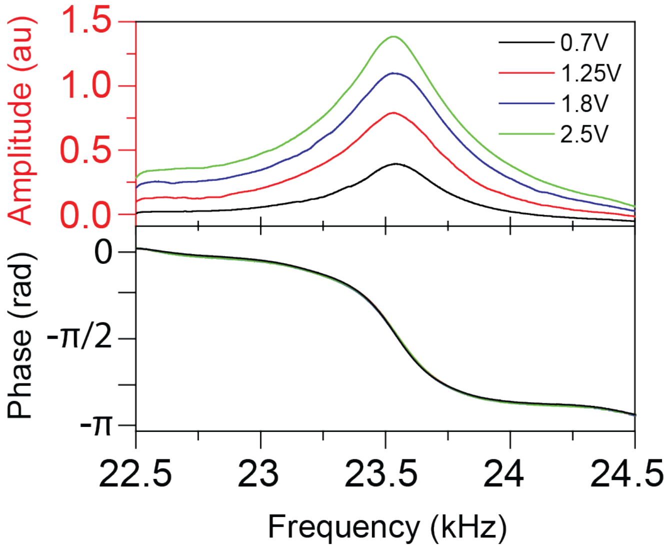

Fig. S 5: Resonance spectra of a PμTR for several different drive amplitudes. Amplitude (top) and phase (bottom) spectra. Four phase spectra are almost identical. The length of the PμTR is 6.5 mm.

Table S 1: Actuation and detection limit of the PμTR-QTF system.

	Actuation (Piezo actuator)	Detection (Piezoelectric readout)
Frequency	$\leq \sim$470 kHz[a]	$\leq \sim$33 kHz[b]
Amplitude	$\leq \sim$2.3 μm[a]	$\geq \sim$30 nm[c]

[a] Resonance frequency and maximum displacement from the manufacturer specification. www.thorlabs.hk/thorproduct.cfm?partnumber=PA4DGW
[b] Resonance frequency from the manufacturer specification. www.abracon.com/Resonators/AB26T.pdf
[c] Equivalent to the noise floor measured and converted by using Fig. S4c

To calibrate the amplitude of the microcapillary in terms of its actual displacement, a soft atomic force microscope (AFM) cantilever (Octo-1000D, Micromotive) was used under a commercial AFM (Nx10, Park Systems). The calibrated stiffness of the Octo-1000D is 0.115 N/m which is three orders of magnitude smaller than that of the microcapillary estimated by equation[2] $4T/d$ ($\sim$200 N/m). The soft AFM cantilever was brought close to and made contact with the mid-point of the microcapillary. When the microcapillary was actuated, electrical signals from the QTF and vibration amplitudes from the position sensitive photo detector[3] (PSPD) in the AFM were measured and recorded simultaneously (Fig. S4a). Calibration results were shown in Fig. S4b for a microcapillary with the length of 6 mm.

The proposed actuation method is asymmetric since the actuation node is offset from the center of mass of the PμTR. To check any problems with this asymmetric actuation, we have measured the vibration amplitude of the microcapillary locally at several points along its longitudinal direction and then have seen symmetric responses (Fig. S4c). Vibration amplitude ratios between the actuation node and the mid-point of the PμTR were found to be $\sim$10 for tested drive voltages (Fig. S4d). In addition, we have not experimentally seen any advantages of our asymmetric actuation method for the asymmetric second flexural mode. In fact, the vibration amplitude of the second flexural mode is much lower than that of the first mode. This is expected since the stiffness for the second flexural mode is higher than that of the first mode and the piezo actuator exhibits resonance frequency (470 kHz) higher than both the first and second modes thus the excitation for both modes would be similar. Interestingly, our actuation method is similar to Melde's experiment which applies excitation at a node and examines the reflection from another node[4].

To check the effect of the drive amplitude on the phase response, we performed additional experiments with a PμTR (Fig. S5). We have not seen any noticeable changes in phase spectra as the drive amplitude was varied. This is because the PμTR was operated in the linear regime. Unless the PμTR is driven to enter the nonlinear regime, its phase response would be independent of the drive amplitude. For our entire measurements, the vibration amplitudes were 0.76 and 4.37 μm, for open and closed loop operations, respectively. Both values are far below the onset of nonlinearity, z_{crit}, calculated to be 13.04 μm by the equation for string resonators[5],

$$z_{crit} \approx 0.56 \frac{d}{\sqrt{Q}} \sqrt{\frac{\sigma}{E}} = 0.56 \frac{d}{\sqrt{Q}} \sqrt{\frac{T}{AE}} \tag{1}$$

where d is the node-to-node distance, σ is the stress, T is the applied tension, and A is the cross-sectional area of the PμTR (σ is equivalent to T/A). Ultimate actuation and detection limits of the PμTR-QTF system in terms of frequency and amplitude are summarized in Table S1.

Several PμTRs were prepared with the node-to-node distance ranging from 4 to 10 mm. After amplitude spectra of PμTRs (Fig. S6a) filled with water were measured, their resonance frequencies and quality factors were extracted by employing the simple harmonic oscillator model[6]. In addition, resonance frequencies were also calculated with the Young's modulus of 62 GPa[7] and the inner and outer diameters of the cross-section of PμTRs of 30 and 50 μm, respectively. Differences between calculated and measured resonance frequencies are within 6% thus showing good agreement. The extracted quality factors of PμTRs range from 120 and 270 (Fig. S6b) which are similar to those of other microfabricated hollow resonators[8] or capillary resonators[9]. Since the air damping is mainly responsible for the relatively low quality factor, further performance improvement of PμTRs is expected once vacuum packaging is employed. Considering efficient QTF's piezoelectric transduction of the vibration of the PμTR, the node-to-node distance of $\sim$6.5 mm which corresponds to the resonance frequency of $\sim$25 kHz is typically used.

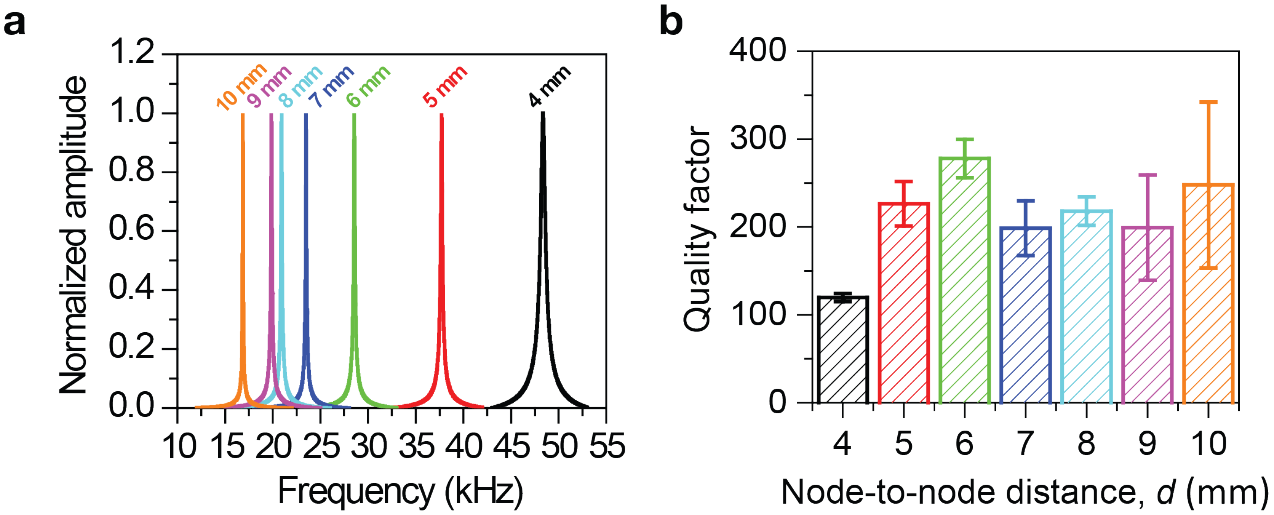

Fig. S 6: **Resonant characteristics of PμTRs. (a)** Amplitude spectra and **(b)** quality factors of PμTRs of which lengths range from 4 to 10 mm. Error bars represent standard errors of the means with $N = 3$.

3 Piezoelectric vs. optical-lever readouts

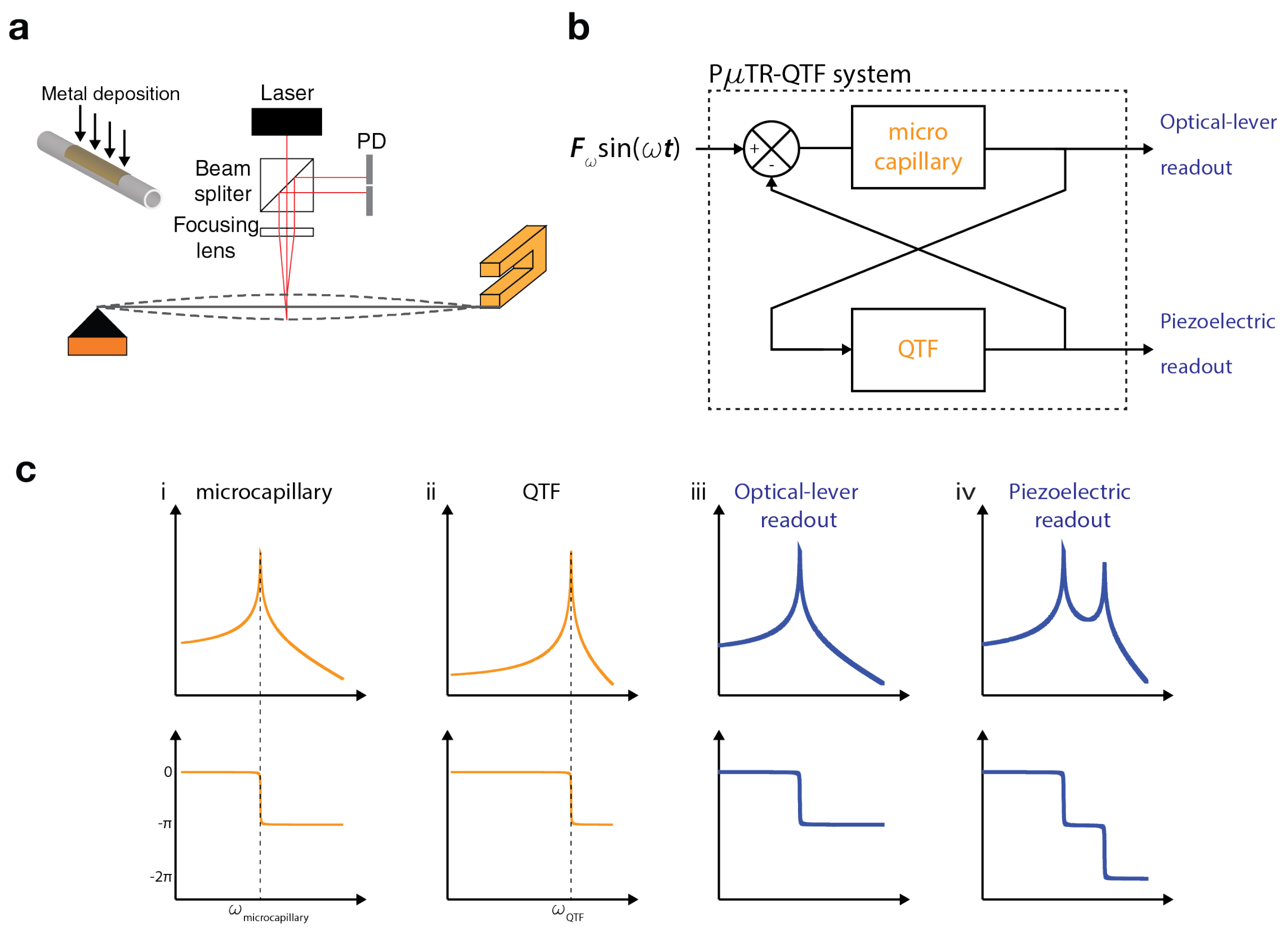

Fig. S 7: Signal transfers in the PμTR-QTF system and its resonance spectra by optical-lever and piezoelectric readouts. (a) Experimental schematic for optical-lever readout of PμTR with partial metal coating. **(b)** Block diagram for the PμTR-QTF system. **(c)** Amplitude and phase spectra of individual components and combined PμTR-QTF system obtained by optical-lever and piezoelectric readouts.

To compare the piezoelectric transduction of the vibration of PμTR, a custom optical-lever setup was built with a continuous laser diode, a beam splitter, a focusing lens, and a two-segmented photodiode as shown in Fig. S7a. Since PμTRs are transparent to the visible range, a thin ($\sim$20 nm) gold layer was partially deposited onto PμTRs by using a shadow mask.

For two readout schemes, signal transfers in the PμTR can be understood by the block diagram (Fig. S7b). Once an external excitation signal vibrates the microcapillary, the vibration of the microcapillary is transmitted to the QTF and the response by the QTF reflects back to the microcapillary. To effectively sense the vibration of the microcapillary by the QTF in both open and closed loop operations, the resonance frequency of the microcapillary is recommended to be lower than but not very close to that of the QTF (Fig. S7c i and ii). When the overall response of the PμTR system is measured by the optical-lever readout, the resonance of the QTF is significantly attenuated by the low-pass filter behaviors of the microcapillary and the transmission loss (Fig. S7c iii). In contrast, the piezoelectric readout can easily capture two resonance behaviors, one for the microcapillary and the other for the QTF (Fig. S7c iv). If PμTR with higher resonance frequencies are required for specific applications, a QTF with a resonance frequency higher than those of PμTR is recommended. Commercial QTFs with resonance frequencies up to $\sim$150 kHz are readily available.

A PμTR with the length of 6.5 mm was measured by both piezoelectric and optical-lever readouts, results were converted into Allan deviations and plotted as a function of the gate time (Fig. S8) or displayed directly in time

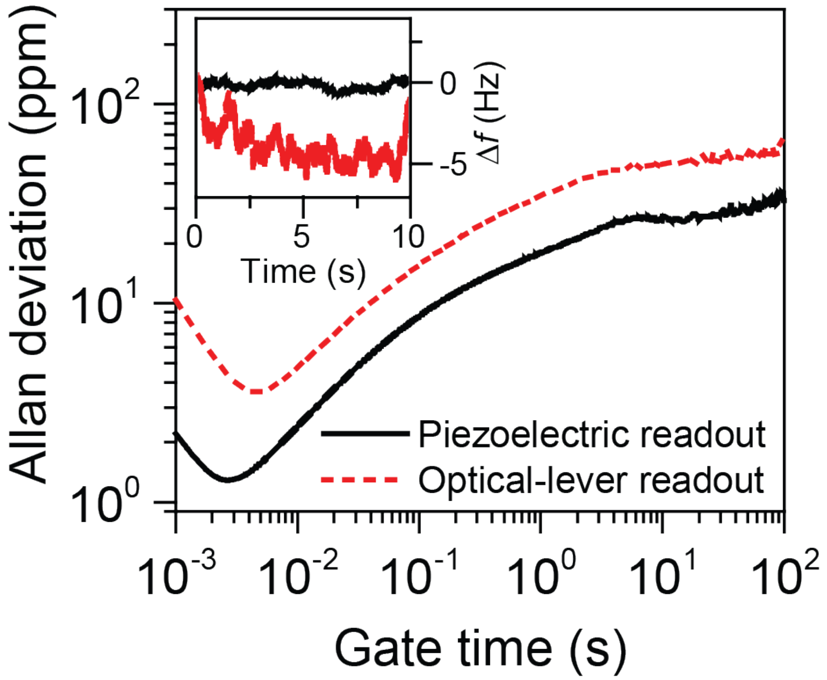

Fig. S 8: Comparison the frequency stabilities between piezoelectric and optical-lever readouts. Allan deviations of the PμTR with the length of 6.5 mm measured with piezoelectric and optical-lever readouts. Inset shows frequency stabilities of the PμTR measured in time domain.

domain (Fig. S8 inset). Both displays indicate that the piezoelectric readout exhibits frequency stability better than the optical-lever readout in our measurements. Such out performance as well as compact and cost-effective setup make the piezoelectric readout ideal for sensing the resonance of PμTRs.

4 Normalized density responsivities of PμTRs

Table S 2: Geometric dimension, tension, and characteristics of three representative PμTRs.

Resonator No.	Length (mm)	Tension (N)	Theoretical f_w (kHz)	Measured f_w (kHz)	Q	R_ρ (Hz-g^{-1}cm^3)
1	6.5	0.3	23.731	25.218	405	3088
2	6.5	0.3	23.731	24.657	307	2754
3	6.0	0.2	20.123	19.901	257	1920

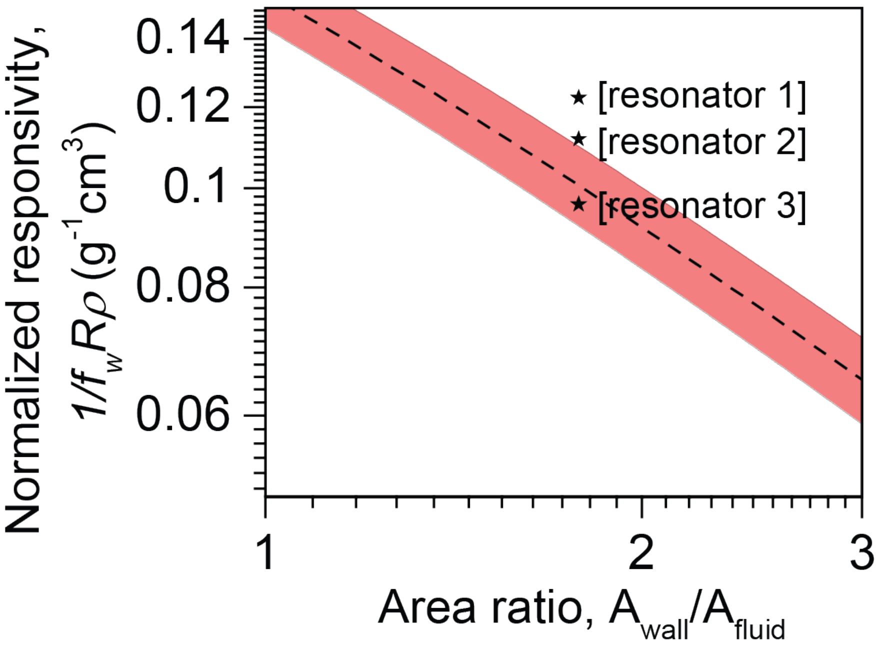

Fig. S 9: **Normalized density responsivity of fabricated PμTRs as a function of the area ratio**

Table S2 summarizes geometric dimensions along with applied tensions, resonance frequencies, quality factors, and density responsivities of three representative PμTRs.

Density responsivities of three PμTRs are also compared in Fig. S9. The PμTR (No. 1 in Table S2) was mainly used for density measurements of binary mixtures and droplet sensing in the main manuscript.

5 Analysis for oil droplet measurements

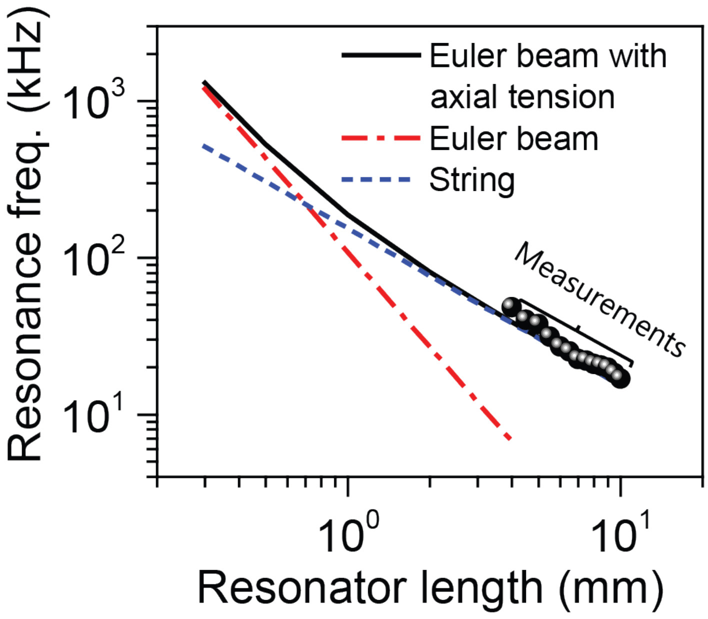

Fig. S 10: **Structural models for PμTRs.** Resonance frequency as a function of the length of resonator based on beam, string, and beam with axial tension models. Measurements for PμTRs of which lengths range from 4 to 10 mm are also shown.

Table S 3: Equations for the resonance frequency based on the three structural models.

	Beam with axial tension	Euler beam	String
Equation	$f_0 = \frac{1}{2d}\sqrt{\frac{T}{m} + \left(\frac{\pi}{d}\right)^2 \frac{EI}{m}}$	$f_0 = \frac{1}{2d}\sqrt{(\frac{\pi}{d})^2 \frac{EI}{m}}$	$f_0 = \frac{1}{2d}\sqrt{\frac{T}{m}}$

Figure S10 shows resonance frequency as a function of the length of resonators exhibiting zero out-of-plane displacement at both ends based on beam, string, and beam with axial tension models (Table S3 for the equation of each model). There is a cross-over between the beam and the string models. In addition, asymptotic limits of the beam with axial tension model approach to the beam model with the length shorter than the cross-over and to the string model with the length longer than the cross-over. Measurements for PμTRs of which lengths range from 4 to 10 mm shown as symbols agree well with the string model. Therefore, the mode shape function U of typical PμTRs (length : ~6.5 mm), included in the following equation for the relative resonance frequency change with the traveling oil plug,

$$\frac{\Delta f}{f_0} = \frac{1}{\sqrt{1 + \frac{m_{op}}{m_r} U^2(x)}} \tag{2}$$

where x is the normalized lateral position of a oil plug along within the PμTR, can be simplified to a single sinusoidal term as seen in Equation (3) in the main manuscript.

Data processing for equivalent radius and velocity of oil plugs is explained thoroughly in Methods section of the main manuscript. Fig. S11 graphically represents the overall processing procedure. The final result of oil droplet data

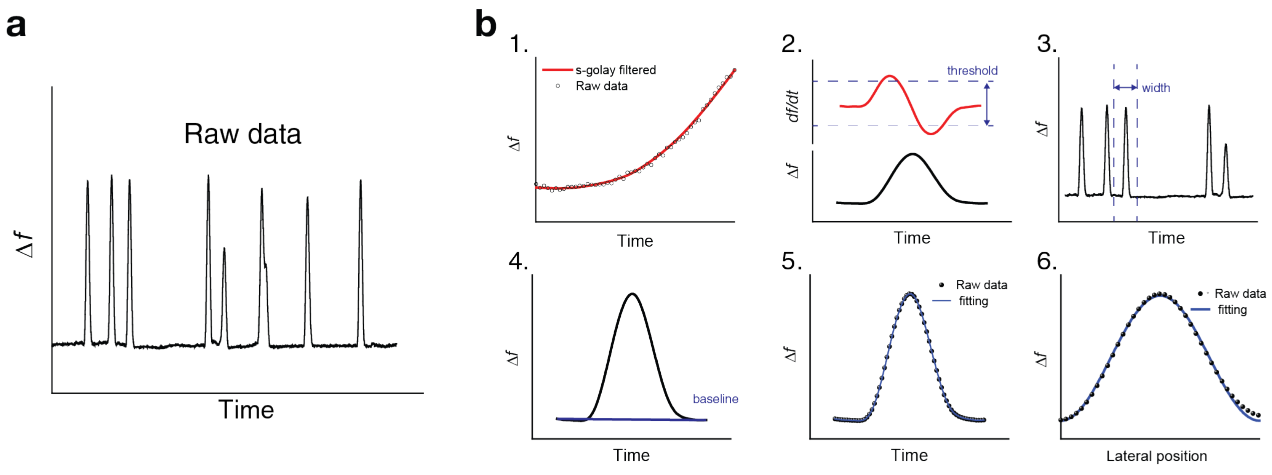

Fig. S 11: Data fitting processing for oil plugs to extract buoyant mass (or equivalent radius) and velocity. **(a)** Frequency change vs. time raw data. **(b)** Step-by-step data processing procedure.

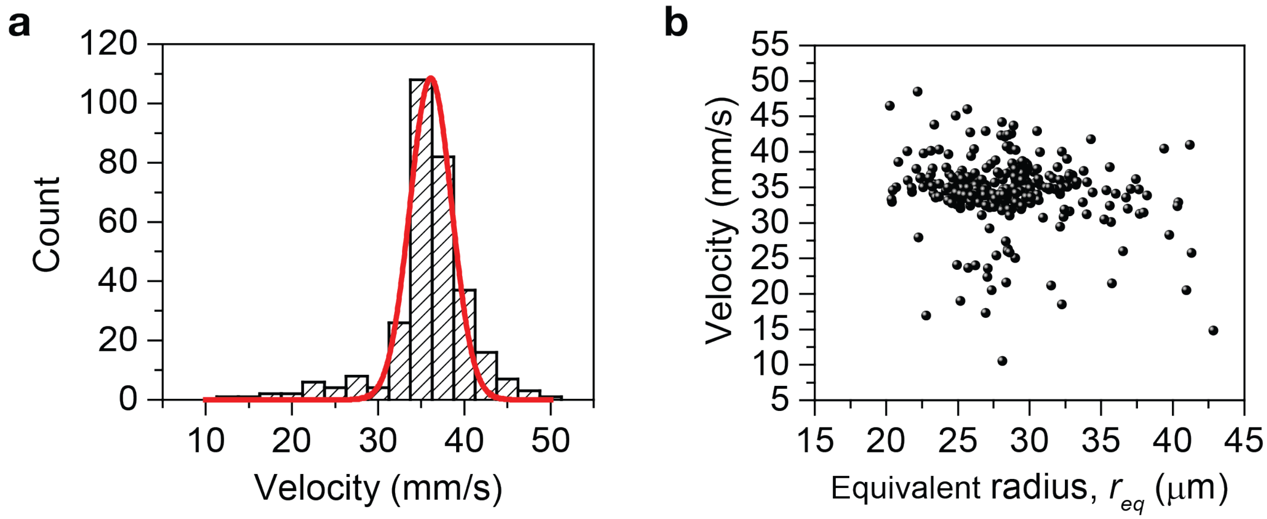

Fig. S 12: Velocity histogram and velocity-radius scatter plot. **(a)** Histogram for the velocity of oil droplets (cumulative counts are 310). **(b)** Scatter plot of velocity versus equivalent radius of oil plugs when pressure difference of 300 kPa is applied.

analysis are shown in Fig. 4f and Fig. S11. Figure S12a shows the velocity histogram for oil plugs passing through the PμTR with the pressure difference of 300 kPa applied. The average velocity of oil plugs is 36.2 mm/s. Figure S12b shows the scatter plot of velocity vs. equivalent radius of oil plugs. No strong correlation between velocity and equivalent radius was observed and this is attributed to multiple oil plugs possibly present in the relatively long pulled section of the microcapillary (∼58 mm) which is ∼9 times longer than the length of the PμTR.

6 Performance tuning for PμTRs

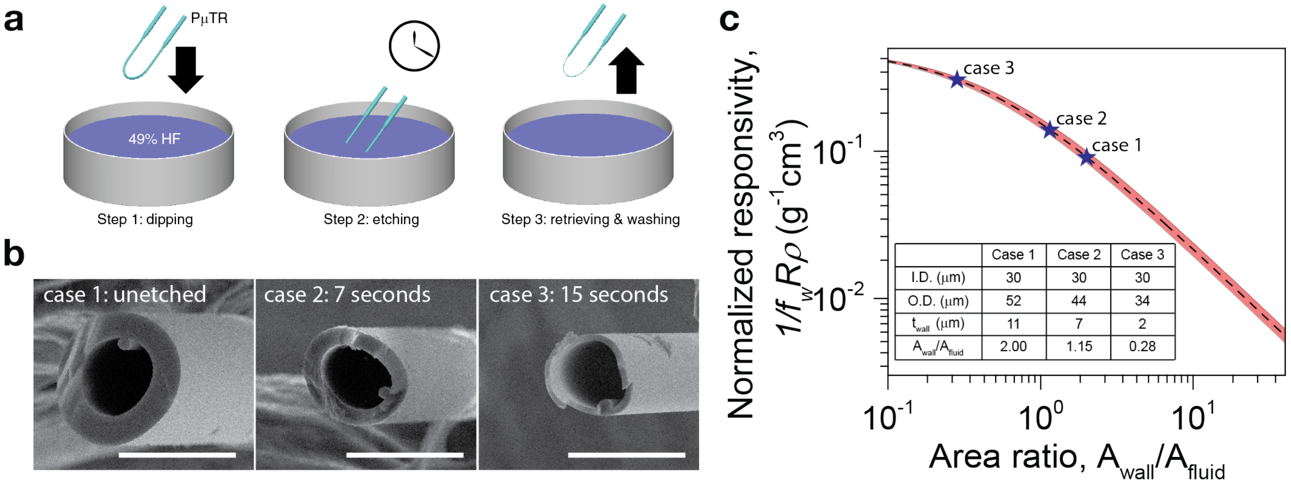

	Case 1	Case 2	Case 3
I.D. (μm)	30	30	30
O.D. (μm)	52	44	34
t_{wall} (μm)	11	7	2
A_{wall}/A_{fluid}	2.00	1.15	0.28

Fig. S 13: **Decreasing the outer diameter (wall thickness) of PμTR via selective hydrofluoric acid etching. (a)** Schematics of the selective etching process by exposing the exterior of the PμTR to hydrofluoric acid. **(b)** Scanning electron micrographs of PμTRs before and after the selective etching. All scale bars are 50 μm. **(c)** Estimated normalized density responsivity of etched PμTRs having a same inner diameter of 30 μm and different wall thicknesses of 2, 7, and 11 μm. Inset table summarizes inner and outer diameters, wall thicknesses, and area ratios of the three PμTRs shown in (b).

Based on Equation (5) in the main manuscript, the normalized density responsivity of a hollow resonator is expected to be improved by decreasing the ratio of A_{wall} to A_{fluid}. Flexible nature of a pulled microcapillary (Figs. 1a and S1) enables the selective etching of its exterior by dipping the pulled region into a hydrofluoric acid (HF) bath with the microcapillary bent 180 degree. After a short period dwell in HF, the microcapillary was taken out, washed in DI water, and dried (Fig. S13a). Scanning electron micrographs show the cross-sections of PμTRs before and after the selective etching process. Wall thicknesses of PμTRs were decreased by 4 and 9 μm after 7 and 15 sec etching, respectively, while their inner diameters were maintained (Fig. S13b). As expected, this selective etching process improves the normalized density responsivity of PμTRs as shown in Fig. S13c. If the microcapillary is entirely dipped into HF, it would be difficult to achieve a uniform inner diameter and there are even possibly etch failures because it requires significant time and effort to wash and remove the HF solution introduced inside the microcapillary. Therefore, it is definitely useful to employ the proposed idea of dipping bent microcapillary into HF which makes the interior of the microcapillary intact. In addition, dilute HFs such as buffered oxide etchants are recommended for better control of the outer diameter (or the area ratio).

There are three important steps for the successful operation of the PμTR after thinning down. First, the etched PμTR is moved and placed on top of the aluminum jig intact and safely. Second, tension (this is force not stress) is applied to the etched PμTR which is then fixed by epoxy gluing. Of note, this is in stark contrast with microfabricated mechanical resonators of which stress (either tensile or compressive) occurs during specific fabrication processes. Third, the QTF is brought close to and makes hard contact with the etched PμTR. During the second step, the maximum allowed tension (limit) is determined to be 0.05 N (Fig. S3) considering the ultimate tensile strength of glass and the cross-sectional area for the most challenging case 3 (wall thickness of 2 μm). To control the tension below the limit is very challenging due to the resolution of the tension gauge limited to 0.02 N. Although the etched PμTR is survived after the step 2, it tends to break during the step 3 due to the hard contact with the QTF. Therefore, we were not able to operate pulled PμTR after selective exterior etching.

Annealing at temperatures around 350 °C which improves the surface roughness of the etched microcapillary and upgrade with a tension gauge with better resolution are recommended to pass up to the step 2. Still, the step 3 would not be favorable as the wall thickness further decreases. In short, the piezoelectric detection with the QTF is not recommended for the etched PμTR. We suggest that the proposed actuation scheme would be paired with the capacitive detection with a remote sensing electrode or with the optical detection which was already used for proof-of-concept experiments.

References

[1] Bray, C. *Dictionary of glass: materials and techniques* (University of Pennsylvania Press, 2001).

[2] Gunther, L. *The physics of music and color* (Springer Science & Business Media, 2011).

[3] Garcia-Sanchez, D. *et al.* Mechanical detection of carbon nanotube resonator vibrations. *Phys. Rev. Lett.* **99**, 085501 (2007).

[4] Tagata, G. Harmonically forced, finite amplitude vibration of a string. *J. Sound Vibration* **51**, 483–492 (1977).

[5] Schmid, S., Villanueva, L. G. & Roukes, M. L. *Fundamentals of Nanomechanical Resonators* (Springer, 2016).

[6] Lee, I. & Lee, J. Measurement uncertainties in resonant characteristics of mems resonators. *J. Mech. Sci. Technol.* **27**, 491–500 (2013).

[7] Bauccio, M. *ASM engineered materials reference book* (CRC, 1994).

[8] Burg, T. P. *et al.* Vacuum-packaged suspended microchannel resonant mass sensor for biomolecular detection. *J. Microelectromech. Syst.* **15**, 1466–1476 (2006).

[9] Malvar, O. *et al.* Highly sensitive measurement of liquid density in air using suspended microcapillary resonators. *Sensors* **15**, 7650–7657 (2015).
